# Brain Tissue Segmentation and Bias Field Correction of MR Image Based on Spatially Coherent FCM with Nonlocal Constraints

**DOI:** 10.1155/2019/4762490

**Published:** 2019-03-03

**Authors:** Jianhua Song, Zhe Zhang

**Affiliations:** ^1^College of Physics and Information Engineering, Minnan Normal University, Zhangzhou 363000, China; ^2^Electronic Engineering College, Heilongjiang University, Harbin 150080, China

## Abstract

Influenced by poor radio frequency field uniformity and gradient-driven eddy currents, intensity inhomogeneity (or bias field) and noise appear in brain magnetic resonance (MR) image. However, some traditional fuzzy c-means clustering algorithms with local spatial constraints often cannot obtain satisfactory segmentation performance. Therefore, an objective function based on spatial coherence for brain MR image segmentation and intensity inhomogeneity correction simultaneously is constructed in this paper. First, a novel similarity measure including local neighboring information is designed to improve the separability of MR data in Gaussian kernel mapping space without image smoothing, and the similarity measure incorporates the spatial distance and grayscale difference between cluster centroid and its neighborhood pixels. Second, the objective function with an adaptive nonlocal spatial regularization term is drawn upon to compensate the drawback of the local spatial information. Meanwhile, bias field information is also embedded into the similarity measure of clustering algorithm. From the comparison between the proposed algorithm and the state-of-the-art methods, our model is more robust to noise in the brain magnetic resonance image, and the bias field is also effectively estimated.

## 1. Introduction

Magnetic resonance image has been widely used in diagnostic imaging for general check-up in clinical application, especially the detection and diagnosis of brain diseases. The volume change for brain tissues often indicates various diseases [1], such as brain tumor, leukoencephalopathy, olivopontocerebellar atrophy (OPCA), etc. Therefore, brain tissue segmentation of MR image has become a very important medical treatment step. However, brain MR image has some lacks such as noise, intensity inhomogeneity, low contrast, the partial volume effect, and so on, which brings serious obstacles to segment the brain MR images. To this end, the multitudinous brain MR image segmentation methods have been put forward by using the theory of fuzzy set, random field, and level set.

Currently, there are two popular methods-based models for medical image segmentation: the random field theory [2–4] and the fuzzy c-means (FCM) algorithm. Random field is density-based unsupervised method where it finds the maximum likelihood estimate of the parameters from a given dataset. However random field algorithm has the disadvantages in high complexity and slow convergence and will drop into local optimization. FCM clustering is another efficient method used in image segmentation because it has robust characteristics for ambiguity and can retain much more information than random field algorithm [5]. Therefore, FCM has been widely applied in different types of image segmentation [6–8]. The neighboring pixels in an image are highly correlated, i.e., the pixels in the immediate neighborhood possess nearly the same feature data. Therefore, the spatial relationship of neighboring pixels is an important characteristic that can be of great aid in imaging segmentation. However, the conventional FCM algorithm does not fully utilize this spatial information. Pedrycz and Waletzky [9] took advantage of the available classified information and actively applied it as part of their optimization procedures. Szilagyi et al. [10] proposed the enhanced FCM (EnFCM) algorithm to accelerate the image segmentation process in which the pixels of an image are replaced the gray-level histogram and the statistical number and calculation are much smaller than FCM. In order to further reduce the computation time and improve the parameter inflexibility, Cai et al. [11] presented a fast generalized FCM (FGFCM) method, and FGFCM introduced a flexible locality factor *S*_*ij*_ incorporating simultaneously both the gray-level difference and spatial distance in a local window. Ji et al. [12] proposed a robust spatially constrained fuzzy c-means (RSCFCM) algorithm for brain MR image segmentation. First, a spatial factor is constructed based on the posterior probabilities and prior probabilities and takes the spatial direction into account. Second, the negative log-posterior is utilized as dissimilarity function by taking the prior probabilities into account.

FCM with spatial constraint and its variants greatly improved the antinoise performance compared with FCM, but when the noise is very serious in the image, the performance of the algorithm may be worse. Therefore, the nonlocal spatial information was often used and incorporated into the distance metric of FCM in recent years [13–16]. Zhao [14] brought in a nonlocal adaptive regularization term in its energy function, and the control factor is adaptive determined to adjust the balance of the objective function. Feng et al. [15] proposed a FCM method with specific nonlocal information for the segmentation of synthetic aperture radar (SAR) image. Ma et al. [16] proposed a modified FGFCM approach by introducing nonlocal constraint term, and local distance metric and nonlocal distance metric are used, respectively, in its objective function. By introducing nonlocal constraint term, the features of image can be used more comprehensively and effectively, and the robustness to noise of FCM-based algorithm is significantly improved. However, there generally exists intensity inhomogeneity in brain MR images. Therefore, it is necessary to further design relevant algorithms to correct the intensity inhomogeneity. Sled et al. designed a set of software package for the estimation of bias field [17], and the characteristic of the method is nonparametric nonuniform intensity normalization or N3 for short; the distribution of the true tissue intensities can be achieved by an iterative method. Tustison et al. [18] improved the N3 algorithm based on modified B-spline approximation and hierarchical optimization algorithm (called N4ITK); N4ITK can also automatically perform without the priori knowledge. Liew and Hong Yan [19] introduced a spatial constraint to a fuzzy cluster method where the inhomogeneity field was modeled by a B-spline surface. The spatial pixel connectivity was implemented by a dissimilarity index, which enforced the connectivity constraint only in the homogeneous areas. Ji et al. proposed the modified possibilistic FCM (MPFCM) algorithm for bias field [20], generalized rough fuzzy c-means (GRFCM) algorithm, [21] and fuzzy local Gaussian mixture model (FLGMM) for brain MR image segmentation [22], respectively. Those methods can estimate bias field and segment the MR images simultaneously.

In this paper, a brain tissue classification and intensity inhomogeneity correction model of MR image based on spatially coherent FCM with nonlocal constraints is proposed. In this model, firstly, both the local constraint term and nonlocal regularization term about brain MR image are incorporated into the objective function, and an adaptive control factor is used to maintain the balance between them. Secondly, the similarity measure is designed in Gaussian kernel mapping space without image filtering, and the detail information and the edge of the image can be preserved well. Meanwhile, bias field model is also embedded into the objective function of clustering algorithm. Therefore, after the intensity inhomogeneity of the MR image is corrected, the segmentation accuracy is improved significantly. Experiments demonstrate that this algorithm can not only effectively estimate the bias field of MR image but also has stronger antinoise ability.

## 2. Preliminary Theory

### 2.1. Fuzzy Clustering Algorithms

Let *X*={*x*_1_, *x*_2_,…, *x*_*N*_} denote an image with *N* pixels, where *x*_*k*_ is gray value of the *k*th pixel in the image. FCM clustering aims at partitioning *X* into *c* clusters by minimizing the following objective function:

(1) $$\begin{array}{c} J_{\text{FCM}}=\overset{c}{\underset{i=1}{\sum }}\overset{N}{\underset{k=1}{\sum }}u_{ik}^{m}{\left\|x_{k}-v_{i}\right\|}^{2}, \end{array}$$

where *v*_*i*_ denotes the *i*th cluster prototype, *u*_*ik*_ denotes the membership degree of *x*_*k*_ belonging to *i*th cluster and follows ∑_*i*=1_^*c*^*u*_*ik*_=1, *c* denotes the number of centers, ‖·‖ denotes the Euclidean norm, and the parameter *m* is a weight exponent on each fuzzy membership that determines the amount of fuzziness of the resulting partition.

Ahmed et al. proposed a modification to FCM objective function by introducing a term that allows the labeling of a pixel to be influenced by the labels in its immediate neighborhood [23]. This effect acts as a regularizer and biases the solution toward piecewise homogeneous labeling. It proved useful in segmenting images corrupted by noise. The modified objective function is given by

(2) $$\begin{array}{c} J_{\text{BCFCM}}=\overset{c}{\underset{i=1}{\sum }}\overset{N}{\underset{k=1}{\sum }}u_{ik}^{m}{\left\|x_{k}-v_{i}\right\|}^{2}+\frac{\alpha }{N_{R}}\overset{c}{\underset{i=1}{\sum }}\overset{N}{\underset{k=1}{\sum }}u_{ik}^{m}\underset{r\in N_{k}}{\sum }{\left\|x_{r}-v_{i}\right\|}^{2}, \end{array}$$

where *x*_*r*_ represents the neighbor voxels of *x*_*k*_ and *N*_*R*_ and *N*_*k*_ stand for the number of voxels in the neighborhood of the *k*th voxel. The parameter *α* controls the intensity of the neighboring effect. One disadvantage of BCFCM is that the neighborhood labeling is computed in each iteration step, something that is very time consuming.

### 2.2. Spatially Coherent Fuzzy c-Means Clustering (SCFCM)

In view of some drawbacks of standard FCM algorithm, a modified scheme is proposed by Despotović et al. [24]. The similarity measure *D*_*ik*_=‖*x*_*k*_ − *v*_*i*_‖^2^ is replaced by *D*_*ik*_^*∗*^=(1 − *S*_*ik*_)‖*x*_*k*_ − *v*_*i*_‖^2^ introducing a weight factor *S*_*ik*_, and the objective function is

(3) $$\begin{array}{c} J_{\text{SCFCM}}=\overset{c}{\underset{i=1}{\sum }}\overset{N}{\underset{k=1}{\sum }}u_{ik}^{m}\left(1-S_{ik}\right){\left\|x_{k}-v_{i}\right\|}^{2}, \end{array}$$

where *S*_*ik*_ is a weight factor including both local spatial information and grayscale difference and is designed as follows:

(4) $$\begin{array}{c} S_{ik}=\frac{\sum_{r\in \Omega_{k}}u_{ir}a_{kr}d_{kr}^{-1}}{\sum_{r\in \Omega_{k}}a_{kr}d_{kr}^{-1}}, \end{array}$$

where *Ω*_*k*_ denotes a local neighboring window around *x*_*k*_, *u*_*ir*_ denotes the membership degree of neighborhood pixels belonging to the *i*th cluster, *a*_*kr*_=|*x*_*k*_ − *x*_*r*_| is the absolute intensity difference between the study pixel and its neighbor, $d_{kr}=\sqrt{{\left(p_{k}-p_{r}\right)}^{2}+{\left(q_{k}-q_{r}\right)}^{2}}$ is the Manhattan distance between the coordinates of pixel *x*_*k*_ and *x*_*r*_, and (*p*_*k*_, *q*_*k*_) and (*p*_*r*_, *q*_*r*_) denote the coordinates *x*_*k*_ and *x*_*r*_ in the image, respectively. By minimizing equation (3) by Lagrangian multiplier approach, *u*_*ik*_ and *v*_*i*_ can be derived as shown in the following equation:

(5) $$\begin{array}{c} u_{ik}=\frac{{\left(\left(1-S_{ik}\right){\left\|x_{k}-v_{i}\right\|}^{2}\right)}^{-1/\left(m-1\right)}}{{\sum_{l=1}^{c}\left(\left(1-S_{lk}\right){\left\|x_{k}-v_{l}\right\|}^{2}\right)}^{-1/\left(m-1\right)}},\\ v_{i}=\frac{\sum_{k=1}^{N}u_{ik}^{m}x_{k}}{\sum_{k=1}^{N}u_{ik}^{m}}. \end{array}$$

Compared with the FCM, this algorithm has two advantages: firstly, it enhances the robustness to all kinds of noise. The constraint item of neighborhood information is included into the similarity measure, so as to effectively utilize the local information of the image. Secondly, it considers anisotropic neighborhood information, and more details and edges information can be preserved. However, the influence of bias field for MR images in segmentation algorithm is not mentioned.

### 2.3. Coherent Local Intensity Clustering Model

Bias field of the MR image usually embodies slowly and smoothly varying for the pixel grayscale of the local region across an image. Meanwhile, in a neighboring local window of the image, bias field can be approximately considered as a constant. Therefore, the most popular model can be described in equation (6) [25]; let *Y* denote the observed image, *b* denote the unknown bias field, *X* denote the true image to be restored, and *n* denote the additive noise.

(6) $$\begin{array}{c} Y=bX+n. \end{array}$$

In the observed image, noise *n* is often assumed to obey Gaussian distribution with zero mean and variance *σ*_*n*_^2^, and the gray level value of the true image approximately takes a constant in a local window. Therefore, the gray level of the observed image can be approximated to obey Gaussian distribution with mean *bX* and variance *σ*_*n*_^2^. In coherent local intensity clustering (CLIC) model [26], a novel metric introducing spatially coherent local intensity convergence criterion for bias field estimation and image segmentation simultaneously is proposed. A Gaussian kernel weight parameter *K*(*r* − *k*) is introduced into the similarity measure of each pixel gray level *x*_*k*_ and its neighbor pixels *x*_*r*_, and the objective function of CLIC is

(7) $$\begin{array}{c} J_{\text{CLIC}}=\overset{c}{\underset{i=1}{\sum }}\overset{N}{\underset{k=1}{\sum }}u_{ik}^{m}\underset{r\in \Omega_{k}}{\sum }K\left(r-k\right){\left\|x_{k}-b_{r}v_{i}\right\|}^{2}, \end{array}$$

where *b*_*r*_*v*_*i*_ is the clustering prototype with bias field in the selective region *Ω*_*k*_, *K*(*r* − *k*) is the weight of a truncated Gaussian kernel allocated for the intensity *x*_*k*_, and the weight parameter can be defined as

(8) $$\begin{array}{c} K\left(y\right)=\left\{\begin{array}{cc} \frac{1}{a}e^{-{\left|y\right|}^{2}/2\sigma^{2}}, & \;\text{for}\;\;\left|y\right|\leq \rho ,\\ 0, & \;\text{else,} \end{array}\right. \end{array}$$

where *σ* denotes the standard deviation, *a* denotes a normalization factor to standardize Gaussian kernel, and *ρ* denotes a radius to measure the size of the local region.

In the CLIC model, intensity inhomogeneity of the MR image can be effectively corrected and can reduce the misclassification rate, but there are some drawbacks in CLIC. When computing the distance metric between the central pixel and its surrounding pixels in a local region, the model only used the local neighborhood information of the pixel without considering the global structure information of the entire image. As a result, the antinoise performance of this algorithm is unsatisfactory.

## 3. Proposed Method

The standard FCM algorithm has the shortcoming of being sensitive to noise. Though, the modified FCM algorithms are improved by adding the spatial information, it is difficult to get a satisfied segmentation result for noise robustness. Therefore, an improved FCM approach based on CLIC and SCFCM is proposed; its objective function is constructed according to the local constraint term and global regularization term; the similarity measure including local neighboring information is designed in Gaussian kernel mapping space, and brain tissue classification and intensity inhomogeneity correction can be realized simultaneously.

### 3.1. Nonlocal Weighted Constraint

In a discrete noisy image *X*={*x*_1_, *x*_2_,…, *x*_*N*_}, *x*_*k*_ is the *k*th pixel and *y*_*k*_ is its nonlocal weighted average value. The derivation method of the nonlocal weighted average can be acquired in [27], and the mathematical expression of *y*_*k*_ is

(9) $$\begin{array}{c} y_{k}=\underset{l\in w_{k}^{u}}{\sum }w_{kl}x_{l}, \end{array}$$

where *w*_*k*_^*u*^ indicates a search region of radius *u* around *x*_*k*_, *w*_*kl*_ denotes nonlocal weight coefficient depending on similarity measure between *x*_*k*_ and its neighboring pixels *x*_*l*_ in window *w*_*k*_^*u*^, and *w*_*kl*_ satisfies the constraint conditions 0 ≤ *w*_*kl*_ ≤ 1 and ∑_*l*_*w*_*kl*_=1. The weight coefficient *w*_*kl*_ is computed as follows:

(10) $$\begin{array}{c} w_{kl}=\frac{1}{Z_{k}}e^{\left(-{\left\|x\left(N_{k}\right)-x\left(N_{l}\right)\right\|}_{2,\sigma }^{2}/h\right)}, \end{array}$$

where *x*(*N*_*k*_) and *x*(*N*_*l*_) denote the grayscale vectors in the square neighborhood *N*_*k*_ and *N*_*l*_ of radius *s* around *x*_*k*_ and *x*_*l*_, respectively, and ‖*x*(*N*_*k*_) − *x*(*N*_*l*_)‖_2,*σ*_^2^ is the weighted Euclidean distance between *x*(*N*_*k*_) and *x*(*N*_*l*_); its expression is defined in equation (11). *σ* is the same as in equation (8), and *h* denotes a control factor to adjust the variation of the similarity measure *w*_*kl*_.

(11) $$\begin{array}{c} {\left\|x\left(N_{k}\right)-x\left(N_{l}\right)\right\|}_{2,\sigma }^{2}=\overset{{\left(2s+1\right)}^{2}}{\underset{p=1}{\sum }}\sigma^{p}{\left(x^{p}\left(N_{k}\right)-x^{p}\left(N_{l}\right)\right)}^{2}, \end{array}$$

where *x*^*p*^(*N*_*k*_) is the *p*th pixel in the grayscale vectors *x*(*N*_*k*_) and *σ*^*p*^ is defined as follows:

(12) $$\begin{array}{c} \sigma^{p}=\overset{s}{\underset{v=\max\left(d,1\right)}{\sum }}\frac{1}{{\left(2v+1\right)}^{2}s}, \end{array}$$

where *y*_*p*_=mod(*p*, (2*s*+1)) and *z*_*p*_=floor(*p*, (2*s*+1))+1, (*y*_*p*_, *z*_*p*_) denote the coordinates of the *p*th component in a preselected region and *d*=max(|*y*_*p*_ − *s* − 1|, |*z*_*p*_ − *s* − 1|).

### 3.2. Objective Function

In order to correct bias field and classify the brain tissues simultaneously, the modified objective function-incorporated local constraint term and nonlocal regularization term is as follows:

(13) $$\begin{array}{c} J_{m}=\overset{c}{\underset{i=1}{\sum }}\overset{N}{\underset{k=1}{\sum }}u_{ik}^{m}\underset{r\in \Omega_{k}}{\sum }K\left(r-k\right)\left[\alpha_{k}\left(1-S_{ik}\right){\left\|x_{k}-b_{k}v_{i}\right\|}^{2}+\left(1-\alpha_{k}\right){\left\|y_{k}-b_{k}v_{i}\right\|}^{2}\right], \end{array}$$

where *u*_*ik*_ is the membership degree of *x*_*k*_ belonging to the *i*th cluster, *Ω*_*k*_ denotes a local square region of the radius *s* around the center *x*_*k*_, *b*_*k*_*v*_*i*_ is the *i*th cluster center in *Ω*_*k*_, *y*_*k*_ denotes the nonlocal weighted average value of *x*_*k*_, *S*_*ik*_ denotes the weight factor of local neighborhood information in equation (4), and the definitions of *K*(*r* − *k*) and *b*_*k*_ are the same as equation (7). *α*_*k*_ is a trade-off weight factor to adjust the balance of local neighborhood information and nonlocal constraints information, and the definition of parameter *α*_*k*_ is

(14) $$\begin{array}{c} \alpha_{k}=\frac{1}{1+\mathrm{var}\left(x\right)/{\left(\bar{x}\right)}^{2}}, \end{array}$$

where $\bar{x}$ denotes the gray level mean of all pixels in the local region *Ω*_*k*_, and var(*x*) denotes the variance of pixel gray values in the same window. The larger the *α*_*k*_ value is, the smaller the influence of the noise is. The factor *α*_*k*_ can be obtained adaptively with the change of the local window *Ω*_*k*_ without being given in advance.

**Theorem 1 .**

Assume ∑_*i*=1_^*c*^*u*_*ik*_=1, 0 ≤ *u*_*ik*_ ≤ 1, and *m* > 1. On the basis of Lagrange multiplier approach, equation (13) is minimized with respect to *u*_*ik*_, *v*_*i*_ and *b*_*k*_ can be derived as shown in the following equation:

(15) $$\begin{array}{c} u_{ik}=\frac{{\sum_{r\in \Omega_{k}}K\left[\alpha_{k}\left(1-S_{ik}\right){\left\|x_{k}-b_{k}v_{i}\right\|}^{2}+\left(1-\alpha_{k}\right){\left\|y_{k}-b_{k}v_{i}\right\|}^{2}\right]}^{-1/\left(m-1\right)}}{\sum_{l=1}^{c}{\left(\sum_{r\in \Omega_{k}}K\left[\alpha_{k}\left(1-S_{lk}\right){\left\|x_{k}-b_{k}v_{l}\right\|}^{2}+\left(1-\alpha_{k}\right){\left\|y_{k}-b_{k}v_{l}\right\|}^{2}\right]\right)}^{-1/\left(m-1\right)}},\\ v_{i}=\frac{\sum_{k=1}^{N}\sum_{r\in \Omega_{k}}Kb_{k}\left[\alpha_{k}\left(1-S_{ik}\right)x_{k}+\left(1-\alpha_{k}\right)y_{k}\right]u_{ik}^{m}}{\sum_{k=1}^{N}\sum_{r\in \Omega_{k}}Kb_{k}^{2}\left(1-\alpha_{k}S_{ik}\right)u_{ik}^{m}},\\ b_{k}=\frac{\sum_{i=1}^{c}\sum_{r\in \Omega_{k}}Kv_{i}\left[\alpha_{k}\left(1-S_{ik}\right)x_{k}+\left(1-\alpha_{k}\right)y_{k}\right]u_{ik}^{m}}{\sum_{i=1}^{c}\sum_{r\in \Omega_{k}}Kv_{i}^{2}\left(1-\alpha_{k}S_{ik}\right)u_{ik}^{m}}. \end{array}$$

**Proof**

According the method of Lagrange multiplier, equation (13) can be converted to unconstrained optimization problem:

(16) $$\begin{array}{c} L\left(u_{ik},v_{i},b_{k},\lambda_{k},\gamma_{k}\right)=\overset{c}{\underset{i=1}{\sum }}\overset{N}{\underset{k=1}{\sum }}u_{ik}^{m}\underset{r\in \Omega_{k}}{\sum }\alpha_{k}K\left(r-k\right)\left[\left(1-S_{ik}\right){\left\|x_{k}-b_{k}v_{i}\right\|}^{2}+\left(1-\alpha_{k}\right){\left\|y_{k}-b_{k}v_{i}\right\|}^{2}\right]+\overset{N}{\underset{k=1}{\sum }}\lambda_{k}\left[1-\overset{c}{\underset{i=1}{\sum }}u_{ik}\right], \end{array}$$

where *λ*_*k*_ is the Lagrange multiplier of the constraint condition ∑_*i*=1_^*c*^*u*_*ik*_=1, by computing the partial derivatives of polynomial *L* in regard to *u*_*ik*_ and *λ*_*k*_, respectively, and set ∂*L*/∂*u*_*ik*_=0, ∂*L*/∂*λ*_*k*_=0, as shown in the following equation:

(17) $$\begin{array}{c} \frac{\partial L}{\partial u_{ik}}=mu_{ik}^{m-1}\underset{r\in \Omega_{k}}{\sum }K\left[\alpha_{k}\left(1-S_{ik}\right){\left\|x_{k}-b_{k}v_{i}\right\|}^{2}+\left(1-\alpha_{k}\right){\left\|y_{k}-b_{k}v_{i}\right\|}^{2}\right]-\lambda_{k}=0, \end{array}$$

(18) $$\begin{array}{c} \frac{\partial L}{\partial \lambda_{k}}=1-\overset{c}{\underset{i=1}{\sum }}u_{ik}=0. \end{array}$$

The following equation can be obtained by mathematical derivation of equation (17):

(19) $$\begin{array}{c} u_{ik}={\left(\frac{\lambda_{k}}{m\sum_{r\in \Omega_{k}}K\left[\alpha_{k}\left(1-S_{ik}\right){\left\|x_{k}-b_{k}v_{i}\right\|}^{2}+\left(1-\alpha_{k}\right){\left\|y_{k}-b_{k}v_{i}\right\|}^{2}\right]}\right)}^{1/m-1}. \end{array}$$

Substituting equation (19) into equation (17), we obtain the following equation:

(20) $$\begin{array}{c} {\left(\frac{\lambda_{k}}{m}\right)}^{1/m-1}={\overset{c}{\underset{i=1}{\sum }}\left(\underset{r\in \Omega_{k}}{\sum }K\left[\alpha_{k}\left(1-S_{ik}\right){\left\|x_{k}-b_{k}v_{i}\right\|}^{2}+\left(1-\alpha_{k}\right){\left\|y_{k}-b_{k}v_{i}\right\|}^{2}\right]\right)}^{-\left(1/m-1\right)}. \end{array}$$

And then substituting equation (20) into equation (19), the following equation can be obtained:

(21) $$\begin{array}{c} u_{ik}=\frac{\sum_{r\in \Omega_{k}}K{\left[\alpha_{k}\left(1-S_{ik}\right){\left\|x_{k}-b_{k}v_{i}\right\|}^{2}+\left(1-\alpha_{k}\right){\left\|y_{k}-b_{k}v_{i}\right\|}^{2}\right]}^{-\left(1/m-1\right)}}{{\sum_{l=1}^{c}\left(\sum_{r\in \Omega_{k}}K\left[\alpha_{k}\left(1-S_{lk}\right){\left\|x_{k}-b_{k}v_{l}\right\|}^{2}+\left(1-\alpha_{k}\right){\left\|y_{k}-b_{k}v_{l}\right\|}^{2}\right]\right)}^{-\left(1/m-1\right)}}. \end{array}$$

Similarly, set ∂*L*/∂*v*_*i*_=0, that is

(22) $$\begin{array}{c} \frac{\partial L}{\partial v_{i}}=\overset{N}{\underset{k=1}{\sum }}u_{ik}^{m}\left(\underset{r\in \Omega_{k}}{\sum }Kb_{k}\left[\alpha_{k}\left(1-S_{ik}\right)\left(x_{k}-b_{k}v_{i}\right)+\left(1-\alpha_{k}\right)\left(y_{k}-b_{k}v_{i}\right)\right]\right)=0. \end{array}$$

The following equation can be obtained from equation (22) by mathematical derivation:

(23) $$\begin{array}{c} v_{i}=\frac{\sum_{k=1}^{N}\sum_{r\in \Omega_{k}}Kb_{k}\left[\alpha_{k}\left(1-S_{ik}\right)x_{k}+\left(1-\alpha_{k}\right)y_{k}\right]u_{ik}^{m}}{\sum_{k=1}^{N}\sum_{r\in \Omega_{k}}Kb_{k}^{2}\left(1-\alpha_{k}S_{ik}\right)u_{ik}^{m}}. \end{array}$$

We adopt the same mathematical derivation process to estimate bias field *b*_*k*_, for fixed *u*_*ik*_ and *λ*_*k*_, and computing the partial derivative of *L* with respect to *b*_*k*_, set ∂*L*/∂*b*_*k*_=0, that is

(24) $$\begin{array}{c} \frac{\partial L}{\partial b_{k}}=\overset{c}{\underset{i=1}{\sum }}u_{ik}^{m}\left(\underset{r\in \Omega_{k}}{\sum }K\left[\alpha_{k}\left(1-S_{ik}\right)\left(v_{i}x_{k}-v_{i}^{2}b_{k}\right)+\left(1-\alpha_{k}\right)\left(v_{i}y_{k}-v_{i}^{2}b_{k}\right)\right]\right)=0, \end{array}$$

*b*_*k*_ can be obtained from equation (24).

(25) $$\begin{array}{c} b_{k}=\frac{\sum_{i=1}^{c}\sum_{r\in \Omega_{k}}Kv_{i}\left[\alpha_{k}\left(1-S_{ik}\right)x_{k}+\left(1-\alpha_{k}\right)y_{k}\right]u_{ik}^{m}}{\sum_{i=1}^{c}\sum_{r\in \Omega_{k}}Kv_{i}^{2}\left(1-\alpha_{k}S_{ik}\right)u_{ik}^{m}}. \end{array}$$

The theorem proves to be completed.

Finally, the framework of the proposed algorithm can be summarized in Table 1.

## 4. Experimental Results and Analysis

In this section, several classical algorithms for intensity inhomogeneity correction and brain image segmentation are selected as the reference for comparison; bias field estimation and antinoise performance analysis for the brain MR images are the main experimental contents. For the experiments in the following sections, the related parameter values are fuzzy exponential *m*=2, the stop criterion *ε*=0.001, 3 × 3 neighborhood window, and the radius *u*=10 of the search window.

### 4.1. Bias Field Correction

#### 4.1.1. MR Image Database

**Figure 1:**
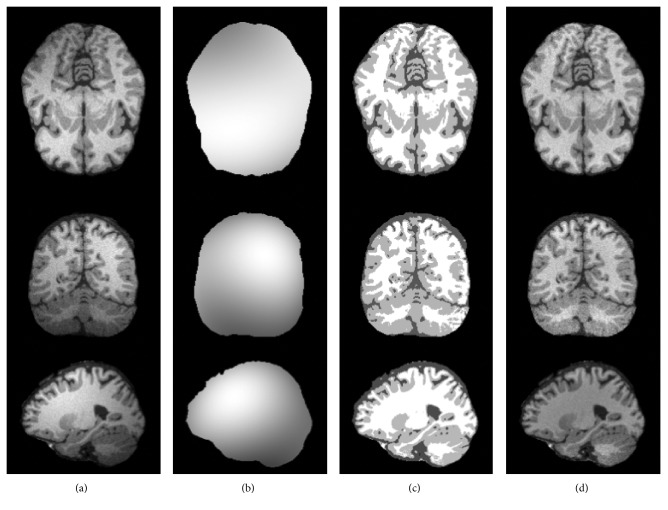
Intensity inhomogeneity correction of brain MR images: (a) original MR images, (b) the estimated bias field, (c) segmentation results, and (d) the corrected MR images.

Intensity inhomogeneity is one of the problems in interfering brain MR image segmentation; in the experiments of bias field correction, the dataset is from a simulated brain database (SBD) : BrainWeb [28] in which the brain MR images have three types: T1-, T2-, and proton density- (PD-) weighted 3D data volumes. In Figure 1, the T1-weighted normal brain MR images with 181 × 217 × 181 cubic voxels, 1 mm slice thickness, 40% intensity nonuniformity, and 3% noise are used to test; all the skulls and blood vessels are already stripped ahead of image processing, and the image is segmented into four regions: white matter (WM), gray matter (GM), cerebrospinal fluid (CSF), and background.

#### 4.1.2. Experimental Results

**Figure 2:**
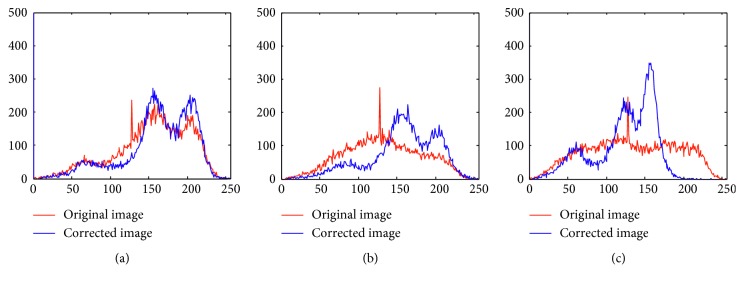
The histogram comparison of original images and corrected images: (a) transaxial slice image, (b) sagittal slice image, and (c) coronal slice image.

Figure 1 shows the results of bias field correction and segmentation for three brain MR images.  Figure 1(a) shows the brain slice images from three different directions: transaxial mode, sagittal mode, and coronal mode.  Figure 1(b) shows the estimated bias field, Figure 1(c) shows the final segmentation results, and Figure 1(d) shows the corrected images after removed bias field.  Figure 2 shows the histogram comparison of original MR image and bias corrected images corresponding to three images in Figure 2. From Figures 1 and 2, three brain tissues are more homogeneous after bias field correction; each brain tissue approximately obeys Gaussian distribution with different mean and variance, and WM, GM, and CSF can be clearly distinguished. In addition, the histogram distribution of corrected MR image is more reasonable, from which we can see three approximative peaks representing three brain tissues.

**Figure 3:**
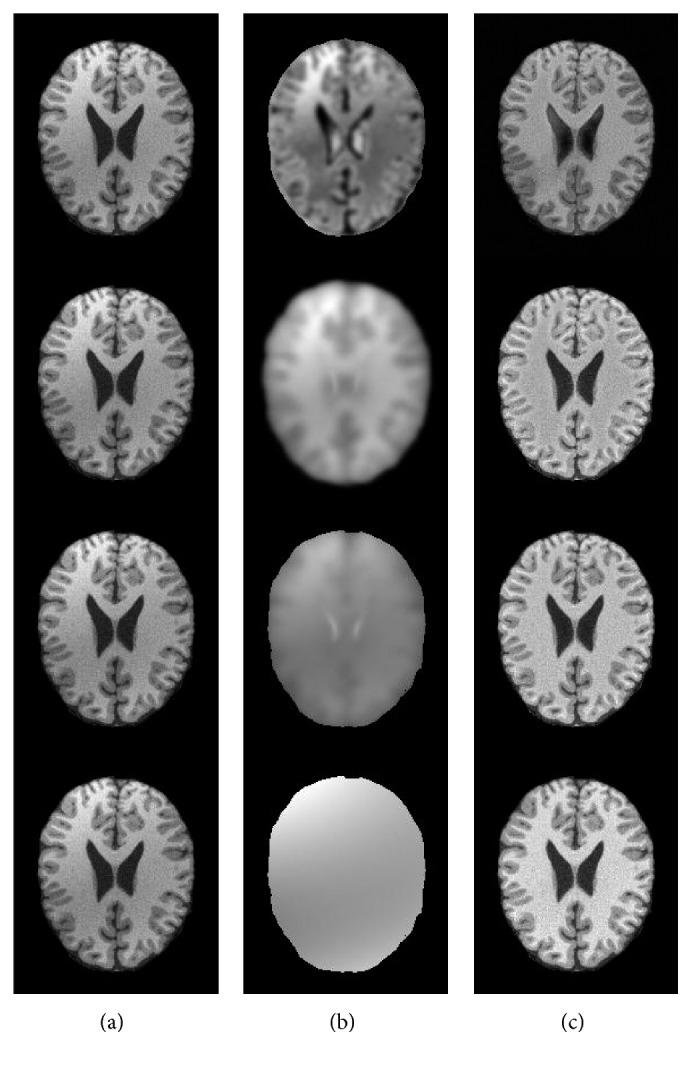
The comparison of bias field estimation by four algorithms: (a) original images, (b) the estimated bias field, and (c) the corrected images.

**Figure 4:**
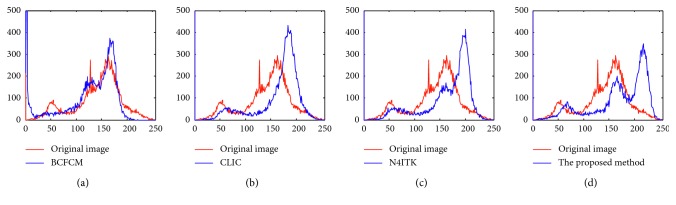
The histogram comparison of original images and corrected images: (a) BCFCM, (b) CLIC, (c) N4ITK, and (d) our method.

To further validate the performance of bias field correction, three bias correction algorithms including BCFCM [23], CLIC [26], and N4ITK [18] are chosen as comparative methods, as shown in Figure 3.  Figure 3(a) is a T1-weighted transaxial slice of normal brain MR image with 40% spatial inhomogeneity; its slice thickness is 1 mm and the noise level is about 2%. Figures 3(b) and 3(c) are the obtained bias inhomogeneity and the amendatory MR images by BCFCM, CLIC, N4ITK, and our method, respectively.  Figure 4 shows the histograms of the image with spatial inhomogeneity and the corrected images in Figure 3. It is can be seen that the distribution of pixel gray level of blue line is more accurate than red dotted line; it indicates that all the algorithms of bias field estimation are more or less effective. However, our method is more reasonable than other three algorithms from Figure 4. Because the histogram normally should have three peaks corresponding to WM, GM, and CSF; the peak value of CSF is minimal according to tissue volume, and the gray level's mean and variance of each tissue are also obviously different.

### 4.2. Antinoise Performance

**Figure 5:**
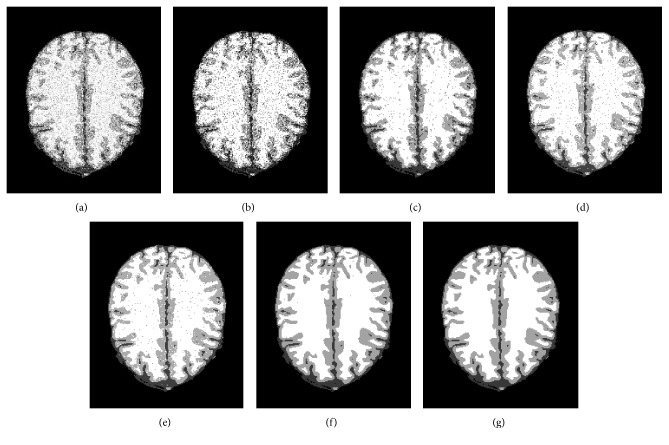
Segmentation results of the 104th transaxial slice by the five algorithms: (a) the noisy MR images, (b) FCM, (c) BCFCM, (d) CLIC, (e) SCFCM, (f) our method, and (g) ground truth.

**Figure 6:**
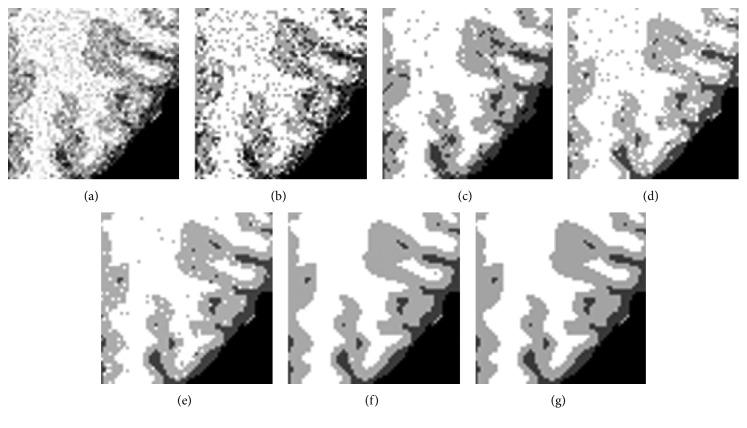
The detailed comparison of the enlarged local region of brain MR image: (a) the noisy MR images, (b) FCM, (c) BCFCM, (d) CLIC, (e) SCFCM, (f) our method, and (g) ground truth.

In the third experiment, first of all, the 104th transaxial slice of simulated T1-weighted brain MR image with 1 mm slice thickness and 7% Gaussian white noise is used to analyze the robustness to the noise, and we select four algorithms: standard FCM, BCFCM [23], CLIC [26], and SCFCM [24] as the compared algorithms. The segmentation results are exhibited in Figure 5; Figure 5(a) is a 2D transaxial slice image corrupted by 7% Gaussian noise, and the corresponding classification results by FCM, BCFCM, CLIC, SCFCM, and the proposed method are shown in the Figures 5(b)–5(f), respectively. The segmentation results of FCM, BCFCM, and CLIC are very poor because many pixels are misclassified; the segmentation result by SCFCM is better than FCM, BCFCM, and CLIC; however, there are still some noisy points that need to be removed. It can be observed that the presented method has more superior segmentation effect than four classical algorithms and can effectively eliminate the influence of the noise. Furthermore, the ability of detail and edge preservation is also compared and analyzed for five algorithms; we select a local region of the MR image to observe by enlarging 3 times, and the detailed images are presented in Figure 6; it is clearly seen that Figure 6(f) is most similar subimage with the ground truth in Figure 6(g), and the vast majority of image details and edge are completely preserved.

**Figure 7:**
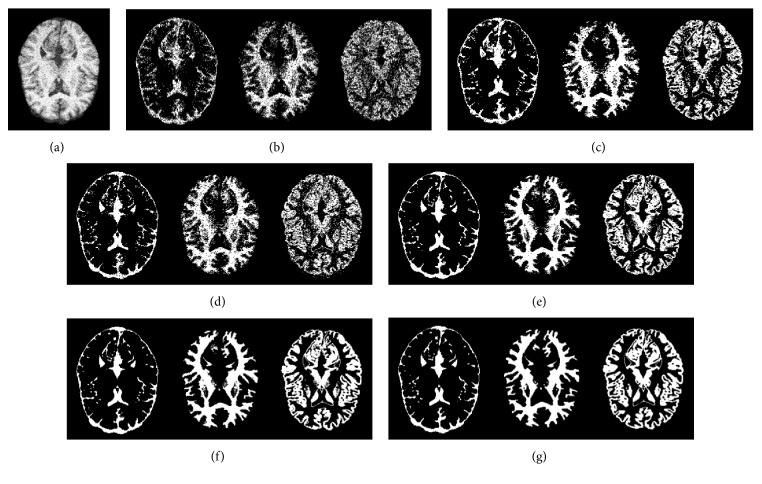
Brain tissue classification results of the five methods on the noisy MR image: (a) original MR image, (b) FCM, (c) BCFCM, (d) CLIC, (e) SCFCM, (f) our method, and (g) ground truth.

**Figure 8:**
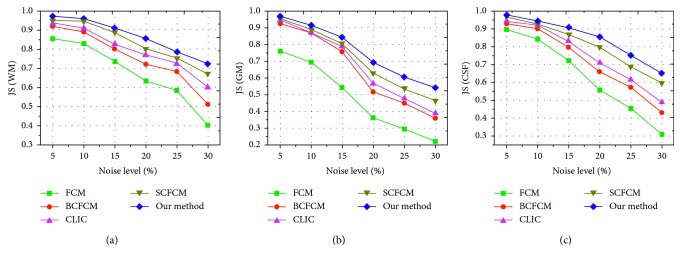
JS comparisons of the brain tissue segmentation with different noise level by five algorithms: (a) JS values of WM, (b) JS values of GM, and (c) JS values of CSF.

In order to further evaluate and compare the antinoise ability of aforementioned five fuzzy clustering algorithms, we choose a brain slice image with 14% additive Gaussian white noise as the test object, as shown in Figure 7.  Figure 7(a) is the noisy MR image with bias field, Figures 7(b)–7(f) are the binary images of CSF, WM, and GM after the image is segmented by five algorithms, respectively, and Figure 7(g) is the ground truth. It is clearly seen that the extraction result of each brain tissue of the proposed algorithm significantly outperformed the other algorithms and effectually overcame the disadvantageous defects of intensity inhomogeneity and noise. At the same time, an objective evaluation index JS (Jaccard similarity) [29] is adopted for comparison and quantitative analysis on the different level noise, JS is given as

(26) $$\begin{array}{c} \text{JS}\left(S_{1},S_{2}\right)=\frac{\left|S_{1}\cap S_{2}\right|}{\left|S_{1}\cup S_{2}\right|}, \end{array}$$

where *S*_1_ represents a set of pixels of the segmented region by a clustering algorithm, *S*_2_ denotes the set of pixels of the corresponding region acquired from the ground truth, ∩ denotes the intersection operation, and ∪ denotes the union operation. As a quantitative evaluation index, the values of JS belong to the interval of [0,1], and the higher the JS value is, the better the segmentation performance is. We selected 15 noisy brain MR images with 20% intensity nonuniformity as the experimental objects and the noise level from 5% to 30%. These images are segmented three regions: WM, GM, and CSF by FCM, BCFCM, CLIC, SCFCM, and our method, respectively; JS values comparison results are shown in Figure 8(a)–8(c). It is clearly shown that the presented approach has better matching degree with the ground truth and higher accuracy rate than other four clustering methods.

**Figure 9:**
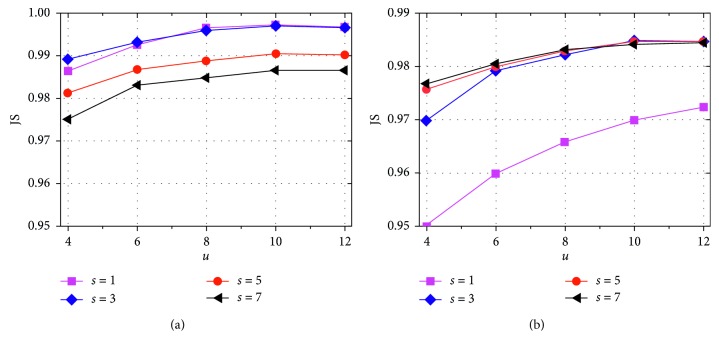
JS value under different search window radius *u* and square neighborhood radius *s*: (a) curves on the image with 1% noise level and (b) curves on the image with 3% noise level.

Then, we evaluate the effect of the search window radius *u* and the square neighborhood radius *s* on the performance of the proposed method. Here, we test *u* and *s* on the sets {4, 6, 8, 10, 12} and {1, 3, 5, 7}, respectively. In this experiment, the tested images perform 8 independent runs of the algorithm under each pair (*u*, *s*), and the noise level is 1% and 3%, respectively. Under each *s* value, the average JS curve of the algorithm with the increase of *u* value is shown in Figure 9. It can be found from Figure 9 that the algorithm under *s* = 3 and *u* = 10 can obtain satisfying performance on the noisy images.

**Figure 10:**
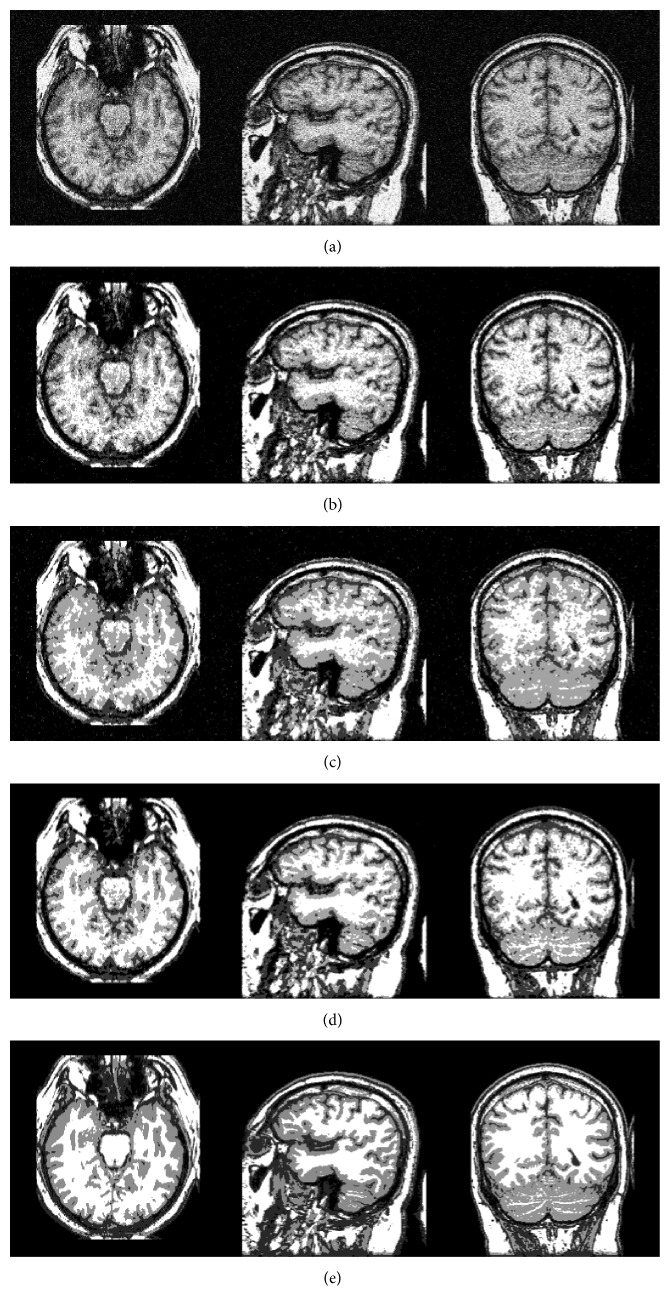
Experimental results on real MR image: (a) three original images, (b) BCFCM, (c) HMRF-EM, (d) SCFCM, and (e) our method.

In the aforementioned sections, the model is applied in the synthetic brain MR images. Next, this model is also applied to the real clinical images with noise. We selected three normal MR slice images from transaxial, coronal, and sagittal views, and these MR images are obtained from the Whole Brain Atlas clinical MR image database by the Harvard medical school [30].  Figure 10(a) shows three 2D T1-weighted brain MR slice images; the left image is a transaxial slice, the right image is a coronal slice, and the middle image is a sagittal slice. The segmentation results of brain slice images are given in Figure 10(b)–10(e) by BCFCM, HMRF-EM, SCFCM, and our method, respectively. From the experimental results, it is obvious that the proposed method can effectively segment each brain tissues as well as preserve more detail information of the original MR image. Furthermore, the experimental results of brain tissues in real MR images also further prove the robustness to noise of the proposed method.

## 5. Conclusion

Brain MR imaging has wide clinical application as an effective medical imaging diagnostic technique; however, the real brain MR images often suffer from some interference such as noise, intensity inhomogeneity, and low contrast. Therefore, a brain tissue classification and nonuniformity field correction scheme in MR images based on spatially coherent FCM with nonlocal constraints is proposed in our study. The available information including local adjacent constraint and nonlocal global information of brain MR image is fully used in our model, and the similarity measure is designed in Gaussian kernel mapping space. Furthermore, the algorithm corrects the bias field of the MR image and improves its antinoise performance. Several experiments on the simulated brain MR images and real brain MR images have demonstrated that the proposed model can effectually overcome the effects of the noise while estimating the bias field existing in brain MR images.

## Figures and Tables

**Table 1:** The basic flow of image segmentation and bias field estimation.

Step 1. Set the number of cluster *c*, the exponent of fuzziness *m*, stopping condition of the iteration *ε* > 0, the radius *s* of local neighborhood window, and the radius *u* of search window in equation (11).
Step 2. Set the iteration initial value *t*=0 and initialize bias field *b*_0_=1 and the center of cluster *V*^1^={*v*_1_^1^, *v*_2_^1^,…, *v*_*c*_^1^}.
Step 3. Calculate nonlocal weight coefficient *w*_*kl*_^*t*^ in equation (8) and then obtain the nonlocal weighted value *y*_*k*_^*t*^ by equation (7).
Step 4. Update the membership degree *u*_*ik*_^*t*^ by equation (14).
Step 5. Update the center of clustering *v*_*i*_^*t*^ by equation (14).
Step 6. Calculate the estimated bias field *b*_*k*_^*t*^ by equation (15).
Step 7. If satisfying max‖*V*^*t*+1^ − *V*^*t*^‖ < *ε*, then terminate iteration; otherwise, go to step 3 and set *t*=*t*+1.

## Data Availability

The data used to support the findings of this study are available from the corresponding author upon request.
